# Pyroptosis: molecular mechanisms and roles in disease

**DOI:** 10.1038/s41422-025-01107-6

**Published:** 2025-04-03

**Authors:** Petr Broz

**Affiliations:** Department of Immunobiology, University of Lausanne, Lausanne, Switzerland

**Keywords:** Innate immunity, Cell death

## Abstract

Pyroptosis is a type of programmed necrosis triggered by the detection of pathogens or endogenous danger signals in the cytosol. Pyroptotic cells exhibit a swollen, enlarged morphology and ultimately undergo lysis, releasing their cytosolic contents — such as proteins, metabolites, and nucleic acids — into the extracellular space. These molecules can function as danger-associated molecular patterns (DAMPs), triggering inflammation when detected by neighboring cells. Mechanistically, pyroptosis is initiated by members of the gasdermin protein family, which were identified a decade ago as pore-forming executors of cell death. Mammalian gasdermins consist of a cytotoxic N-terminal domain, a flexible linker, and a C-terminal regulatory domain that binds to and inhibits the N-terminus. Proteolytic cleavage within the linker releases the N-terminal domain, enabling it to target various cellular membranes, including nuclear, mitochondrial, and plasma membranes, where it forms large transmembrane pores. Gasdermin pores in the plasma membrane disrupt the electrochemical gradient, leading to water influx and cell swelling. Their formation also activates the membrane protein ninjurin-1 (NINJ1), which oligomerizes to drive complete plasma membrane rupture and the release of large DAMPs. Since their discovery as pore-forming proteins, gasdermins have been linked to pyroptosis not only in host defense but also in various pathological conditions. This review explores the history of pyroptosis, recent insights into gasdermin activation, the cellular consequences of pore formation, and the physiological roles of pyroptosis.

## Introduction

Cell death is an essential process that is required for the development of multicellular organisms, the maintenance of tissue homeostasis, as well as for immunity and host defense.^1^ The importance of programmed cell death (PCD) is highlighted by the fact that animals with deficiencies in signaling pathways that regulate the induction of cell death die during embryogenesis, display developmental defects, or have a higher susceptibility to infection.^2^ Cell death was initially classified into two forms: an actively executed or regulated form of cell death called apoptosis, and passive, unregulated lysis of cells caused by external factors (e.g., cellular damage or toxins) called necrosis.^3^ Apoptosis is initiated by specific signaling cascades that regulate the activation of apoptotic caspases, a family of cysteine proteases that execute cell death, and is thus classified as a PCD.^1,2^ Apoptosis preserves plasma membrane integrity and is thus generally considered to be immunologically silent, whereas plasma membrane rupture (PMR) during necrosis releases cytosolic factors that act as so-called ‘danger signals’ or ‘danger-associated molecular patterns (DAMPs)’,^4^ i.e., molecules that drive inflammation in tissues when detected by neighboring cells.

Research over the past two decades has blurred the distinction between PCD and necrosis, since several additional cell death modalities have been identified that all result in a programmed or regulated form of necrosis.^2^ The best-studied among these modalities are pyroptosis, necroptosis and ferroptosis. In this review, I focus on pyroptosis, a lytic form of cell death first reported in the late 1990s as part of the immune response to pathogen infection.^5^ Pyroptosis is primarily linked to the activation of inflammasome complexes, which are innate immune signaling platforms assembled by pattern recognition receptors (PRRs) upon the detection of pathogen-derived or endogenous danger signals.^6^ However, since the discovery of the gasdermins^7^ — a family of pore-forming proteins that initiate pyroptosis — it has been recognized that pyroptosis can also be induced by many other signaling pathways independent of inflammasome activation. This has led to a rapid expansion of pyroptosis research revealing important functions of this form of cell death in immunity, development and cancer. Here, I summarize the mechanisms that induce pyroptosis at a cellular level and its role in disease, with a special focus on recent insights into the molecular mechanisms that regulate gasdermin activation and the induction of cell death.

## Pyroptosis and the discovery of the gasdermins

**Fig. 1:**
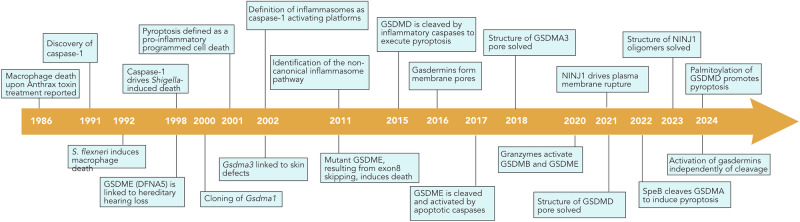
Key events in the history of pyroptosis. Timeline listing major discoveries since the first reports related to pyroptosis.

First reports of pyroptosis date back to the early 1990s, when it was noted that *Bacillus anthracis* toxin or *Shigella flexneri* infections result in macrophage cell death (Fig. 1).^8,9^ Since DNA fragmentation could readily be detected in *S. flexneri*-infected cells, it was initially assumed that this type of cell death was apoptosis.^9^ Later studies found that this form of cell death was also induced by other microbes,^10–12^ and that it involved DNA fragmentation without the typical nuclear condensation observed in apoptotic cells. Furthermore, this form of cell death resulted in permeabilization of the plasma membrane and necrotic morphology, inconsistent with apoptosis induction.^13^ Clear evidence that this novel form of programmed necrosis was distinct from apoptosis came from findings showing that its induction did not require apoptotic caspases, but rather relied on caspase-1, an inflammatory caspase.^14,15^ Caspase-1 had been previously identified as the interleukin (IL)-1-converting enzyme (ICE), which cleaved pro-IL-1β/18 to their mature secreted forms.^16^ Consistent with this, it was also found that the caspase-1-dependent necrosis correlated with the release of mature, bioactive IL-1β/-18.^17^ To clearly distinguish this novel form of death from accidental necrosis or programmed apoptosis, the term pyroptosis was proposed in 2001, stemming from the Greek *pyr*o (meaning fire or fever) and *ptosis* (meaning to fall), thus underlining the proinflammatory nature of this form of cell death.^5^

Coining the term pyroptosis coincided with the molecular characterization of the inflammasome — a platform for the activation of caspase-1.^18^ Inflammasomes are signaling complexes that are assembled by cytosolic PRRs in response to infection, injury, or noxious substances.^6^ ‘Canonical’ inflammasomes activate caspase-1 and are assembled by the proteins Pyrin, AIM2, CARD8, and multiple members of the NOD-like receptor (NLR) family, which directly or indirectly detect pathogen-derived signals (pathogen-associated molecular patterns, PAMPs) or endogenous danger signals (DAMPs). Conversely, the non-canonical inflammasome pathway, which was first reported in 2011,^19^ controls the activation of caspase-11 in mice or caspases-4/-5 in humans in response to the bacterial cell wall component lipopolysaccharide (LPS).^20,21^ Since activation of inflammasomes not only led to caspase-1-dependent production of IL-1β/-18, but also, in most cases, to a caspase-dependent cell death, pyroptosis was redefined as an inflammasome-dependent cell death and an effector mechanism of the inflammasome.^22^ Nevertheless, how active caspase-1, -11, or -4 induced cell death remained unknown for another decade. But the fact that it required the proteolytic activity of caspases suggested that cleavage of one or several yet unidentified substrate/s must initiate pyroptosis.

Landmark studies published in 2015 finally reported that a single caspase substrate, called gasdermin D (GSDMD), was the long-sought after executioner of pyroptosis.^23–25^ GSDMD is a 53 kDa cytosolic protein and a member of the gasdermin protein family, which features 6 members in humans (GSDMA, -B, -C, -D, -E and -F (also known as pejvakin, PJVK), and 10 in mice (GSDMA1-3, GSDMC1-4, GSDMD, GSDME and PVJK).^7^ Gasdermins were first identified in the early 2000s and named after the expression pattern of the first family member, GSDMA, in the gastrointestinal tract and the dermis.^26^ Although links to inflammation and cell death had emerged over time,^27–29^ their function as cell death executioners had not been previously recognized. All three studies that identified GSDMD as the executioner of inflammasome-associated pyroptosis found that *GSDMD*-deficient cells showed a significant reduction in pyroptosis induction after activation of both canonical and non-canonical inflammasomes.^23–25^ They also demonstrated that caspase-1, -11, or -4 cleave GSDMD after an aspartate residue within a central linker domain (D275 in humans and D276 in mice) to generate a 31-kDa N-terminal GSDMD fragment (GSDMD-NT) and a 22-kDa C-terminal fragment (GSDMD-CT). Furthermore, it was found that expression of the GSDMD-NT induced cytotoxicity with all features of pyroptosis, while the expression of full-length GSDMD (GSDMD-FL) or GSDMD-CT had no impact on cell viability, supporting the conclusion that caspase-mediated cleavage releases the cytotoxic GSDMD-NT from intramolecular autoinhibition by GSDMD-CT.

These findings revealed that the characteristic two-domain structure, comprising an N-terminal cytotoxic domain and a C-terminal inhibitory domain linked by a connector, is not unique to GSDMD but is shared by all gasdermin family members, except for PJVK, which has a truncated C-terminal domain. Moreover, ectopic expression of the N-terminal domain of GSDMA, GSDMB, GSDMC, or GSDME triggers a form of cellular necrosis that resembles pyroptosis induced by GSDMD.^30^ Consequently, soon after GSDMD was identified as the executor of inflammasome-associated pyroptosis, the gasdermin family was recognized as a novel group of cell death effectors defined by their N-terminal pyroptosis-inducing domain (GSDM-NT).

Additionally, since other gasdermin family members can be activated independently of inflammatory caspases,^31^ these discoveries indicated that pyroptosis is not solely a consequence of inflammasome activation. This necessitated a reclassification of pyroptosis as a form of mammalian cell death mediated by gasdermin family members.^7^

## Pore formation constitutes the mechanism of gasdermin-induced pyroptosis

**Fig. 2:**
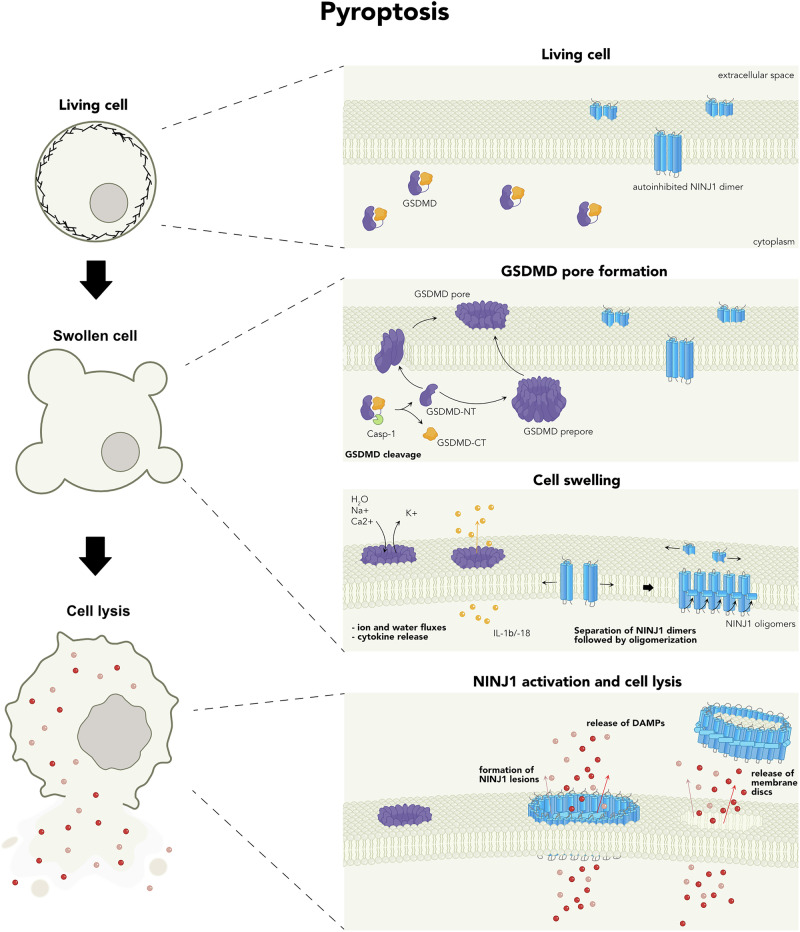
Sequence of events during inflammasome-activated pyroptosis. Caspase-1 cleaves GSDMD, separating the GSDMD-NT and GSDMD-CT domains. This cleavage relieves the autoinhibition of GSDMD-NT, enabling it to interact with and insert into the plasma membrane and progressively form pores, or to form pre-pores that insert into the membrane. Once GSDMD pores are formed, ion and water fluxes lead to cell swelling. Mature cytokines, such as IL-1β and IL-18, can be released directly through these pores. An as-yet unidentified signal activates NINJ1, which exists in the plasma membrane as autoinhibited dimers during the swelling phase. This activation causes a conformational change in alpha helix 1 of NINJ1, leading to the formation of flexible, filamentous NINJ1 oligomers. These oligomers can bend to form pore-like structures or release membrane discs via their hydrophilic surface. The resulting NINJ1 pores or lesions facilitate the release of DAMPs, completing the process of pyroptosis.

Several follow-up studies investigated the mechanism of GSDMD-NT-induced cell death using a combination of biochemistry, cell biology and structural biology (Fig. 2). These studies found that upon cleavage, GSDMD-NT associates with cellular membranes, and that it preferentially targets acidic phospholipids such as phosphoinositides that are found on the inner leaflet of the plasma membrane. The N-terminal domains of other gasdermins, such as those of GSDME, GSDMA and murine GSDMA3, exhibit similar lipid-binding properties, suggesting a common membrane-targeting mechanism for the entire gasdermin family.^30,32^ Cardiolipin was also found to be a strong binder of GSDM-NT, indicating that gasdermins might also target mitochondrial or bacterial membranes that contain cardiolipin. Consistently, it was recently reported that GSDMD targets and permeabilizes mitochondria in a manner requiring cardiolipin.^33^ Liposome binding assays further confirmed that GSDMD-NT interacted with membranes, and in addition it was noted that upon binding, the GSDMD-NT permeabilized liposomal membranes.^30,32,34,35^ Finally, atomic force microscopy or electron microscopy analysis of these liposomes revealed that GSDMD-NT forms pore-like structures in the membranes with an average inner diameter of around 20 nm.^30,32,34,35^ In summary, these studies thus demonstrated that gasdermin family members, with the exception of PJVK, constitute a novel family of pore-forming cell death executioners.

Today, the mechanism of gasdermin autoinhibition and pore formation has been well characterized at the molecular level, and high-resolution cryo-EM structures of pores formed by GSDMA3, GSDMD and GSDMB have been published.^36–38^ The structures of full-length mGSDMA3 and m/hGSDMD, for example, show that the GSDM-NT consists of a twisted β-sheet that is surrounded by α-helices and a disordered region that is involved in membrane insertion during pore formation.^39,40^ By contrast, the GSDM-CT consists primarily of tightly packed α-helices.^30^ In the full-length protein, the GSDM-CT binds and masks membrane-binding elements in GSDM-NT, thus providing the molecular basis for GSDM autoinhibition.^30,39,40^ The main mechanism that can relieve this autoinhibition and promote GSDM activation is proteolytic cleavage that separates the N- and C-terminal parts, even though other mechanisms are emerging as well, as discussed later. Studies have shown that to process GSDMD, inflammatory caspases first interact with an exosite within the GSDMD-CT,^41^ which helps to position the interdomain cleavage site of GSDMD close to the catalytic site of the caspases. Interestingly, exosite binding determines substrate specificity but not the tetrapeptide motif (FLTD in human GSDMD), even though Asp275/276 (human/mouse) remains important for cleavage. A similar mechanism applies to the cleavage of pro-IL-18 by caspase-4,^42,43^ suggesting a generalized mechanism for the cleavage of substrates of inflammatory caspases. Whether GSDMD cleavage can be inhibited by targeting exosite recognition, and which mechanisms apply to the cleavage of other gasdermin family members by other proteases, remains to be determined.

Structural studies on pores formed by recombinant gasdermin N-termini have shown that membrane-inserted GSDM pores feature a coronary ring and a transmembrane β-barrel ring, organized by the large globular subdomain and two side-by-side extended β-hairpins of the GSDM-NT domain.^36–38^ The pore is formed by either 26–28 (GSDMA3), 26–30 (GSDMB) or 31–34 (GSDMD) protomers. This gives rise to pores with an internal diameter of 18 nm, 16 nm or 21.5 nm, respectively. These studies also revealed non-β-barrel pore structures, possibly hinting at the existence of pre-pore structures.^36^ Comparison of the GSDM-NT in its full-length and pore conformations shows that the protein undergoes major conformational changes during pore formation and assumes the shape of a hand, with the membrane-binding α-helix 1 as a thumb, two β-hairpins as fingers and the rest of the protein forming the palm. Membrane interaction involves two β-hairpins from each protomer that form the β-barrel, and the positively charged helix α1, which is involved in the interaction with negatively charged head groups of phospholipids on the inner leaflet of the plasma membrane. In the autoinhibited, un-cleaved form of the protein, helix α1 and the α1–α2 loops are masked by the GSDM-CT, preventing premature membrane interaction.

## Ninjurin-1 is a downstream effector of gasdermin pore formation

Early descriptions of pyroptosis postulated that pyroptosis involved the initial formation of a small pore that drove ion fluxes, followed by oncotic swelling that eventually resulted in plasma membrane rupture (PMR) and passive cell lysis.^44^ Interestingly, a recent report showed that PMR is actively executed by a small 16-kDa plasma membrane protein known as ninjurin-1 (NINJ1).^45^ In the absence of NINJ1, cells still induce GSDMD cleavage and pore formation, as measured by the uptake of small DNA-binding dye such as propidium iodide (PI), but fail to release lactate dehydrogenase (LDH), which has often been used as a readout for pyroptosis induction. Furthermore, NINJ1-deficient cells still release IL-1β and IL-18 upon inflammasome activation via GSDMD pores, demonstrating that in the absence of cell lysis, GSDMD pores can serve as direct channels for protein secretion. NINJ1 deficiency does, however, not protect cells from death but efficiently blocks the release of lysis-associated DAMPs like HMGB1 and S100 proteins, suggesting that pyroptosis induction might be less pro-inflammatory in the absence of NINJ1.

The signal that activates NINJ1 is currently unknown, and, although likely, it remains unknown whether all gasdermins can trigger NINJ1 activation. Since NINJ1 activation was observed during almost all forms of necrotic death, ranging from toxin-induced necrosis to ferroptosis and cuproptosis,^46–48^ and even when apoptotic cells undergo secondary necrosis,^45^ it can be assumed that NINJ1 senses a universal signal that is produced when cells undergo necrosis, possibly linked to changing plasma membrane properties such as tension, fluidity, lipid asymmetry or lipid distribution.

Upon sensing its activation signal, NINJ1 starts to form higher-order oligomers that were recently characterized by super-resolution microscopy and cryo-EM,^48–50^ shedding light on how NINJ1 permeabilizes the membrane. A first study reporting the cryo-EM high-resolution structure of active NINJ1 showed that the protein forms flexible filaments with both a hydrophobic and a hydrophilic face, where NINJ1 protomers are tightly packed in a fence-like array of transmembrane α-helices.^48^ The structured part of each protomer comprises 4 α-helices, of which α-3 and α-4 are fully extended to pass through the membrane, while α-1 and α-2 form a kinked helix, in which helix α-1 crosses over from one protomer to the next to stabilize the filament. The fact that NINJ1 filaments have a hydrophilic and hydrophobic face suggests that they can cap and stabilize membrane edges, forming large transmembrane lesions (Fig. 2).^48^ Alternatively, membrane permeabilization could involve the formation of double filaments that associate via their hydrophilic faces, followed by a gradual zipper-like opening once cells swell. Two later studies report a very similar structure with a kinked α1–α2 helix, but a slightly bent α-3 and α-4 helix.^49,50^ They also find that the NINJ1 filament is slightly bent, with the concave side being hydrophobic and the convex side being hydrophilic. Based on this, the studies propose that NINJ1 forms lesions via a ‘membrane-loss’ model, in which NINJ1 oligomers solubilize membrane patches by encircling them (Fig. 2). Additional studies involving the visualization of NINJ1-induced lesions in cells will be necessary to define whether one or both models apply to NINJ1-driven PMR. Possibly a better understanding of NINJ2, a homolog of NINJ1, could help as well, since NINJ2 was found to also form similar filaments in vitro,^49,50^ but lacks the ability to permeabilize cells.^45,51^

## Morphological features of pyroptosis

All forms of cell death result in pronounced biochemical, metabolic, and morphological changes at the cellular level. Early studies noted that pyroptotic cells display several striking morphological features that make them distinct from the morphology of apoptotic cells, which are characterized by pyknosis (shrinkage or condensation of a cell with increased nuclear compactness or density), membrane blebbing, and the formation of distinct apoptotic bodies.^52^ Furthermore, apoptotic cells maintain plasma membrane integrity. By contrast, pyroptosis starts with a loss of plasma membrane integrity due to gasdermin pore formation, resulting in non-specific fluxes of calcium, sodium, and potassium ions, thereby dissipating the electrochemical gradient (Fig. 2).^53^ Gasdermin pore formation is followed by cell rounding and swelling, culminating usually in the formation of one or several large membrane balloons (Fig. 2). During this process, pyroptotic cells also undergo nuclear rounding and condensation and suffer decay of mitochondria and other organelles, which will eventually lead to metabolic cell death.^54^ Pyroptotic cells also expose phosphatidylserine (PS) at the plasma membrane, potentially caused by membrane scrambling mediated by TMEM16F, which is activated by calcium fluxes.^55^ Finally, these events culminate in NINJ1-dependent loss of plasma membrane integrity, causing the cells to lose their swollen morphology.^45^ At this stage, large lesions are formed by NINJ1, either by the formation of NINJ1 pores or by the release of membrane discs.^48–50^ Mass spectrometry studies have shown that cells release cytosolic and organellar proteins in an NINJ1-dependent manner, which can act as potential danger signals.^45,46^ A recent work also found that NINJ1 lesions are needed to allow filamentous actin from necrotic cells to engage CLEC9A signaling in dendritic cells.^56^ Experimentally, the easiest readout for NINJ1-induced cell lysis is to measure the release of LDH from cells or the uptake of large dyes. It is worth noting that in the absence of NINJ1, pyroptosis will proceed to the stage of cell swelling, and the cell will be dead and will take up PI or other dyes via GSDM pores. Thus, DNA-binding dyes are not well-suited for assessing NINJ1 lesion formation. After their demise, pyroptotic cells have also been reported to release extracellular vesicles (EVs) that can deliver signals to distant cells.^57–59^ EVs might be generated by membrane repair mechanisms, such as ESCRT, that remove GSDMD pores from membranes^60^ or by other processes.

All the above changes are initiated by the formation of gasdermin pores, and can be recapitulated in cells with inducible expression of GSDM-NT, and are thus independent of the upstream caspases that cleave and activate gasdermins. However, in the context of inflammasome activation, for example, inflammatory caspases cleave not only GSDMD but also pro-IL-1β and -18.^6^ Interestingly, the mature form — but not the precursors — is released from pyroptotic cells independently of cell lysis as shown by the use of osmoprotectants that prevent lysis or genetically by deleting NINJ1.^37,45,61^ This indicates that GSDM pore formation might be associated with the limited release of cytosolic proteins even before full permeabilization, yet, thus far, this pore-specific secretome has not been described or functionally characterized. Two recent studies reported that in addition to proteins, oxylipins and metabolites can also be released from pyroptotic cells, and that these can drive tissue repair.^62,63^ Furthermore, under certain conditions, inflammasome activation can induce IL-1β release without pyroptotic cell death,^64^ a phenomenon called hyperactivation. This suggests that GSDM pore formation may facilitate unconventional protein secretion independent of cell death.^65^

Many studies on pyroptosis primarily use mouse or human macrophages, but this form of cell death may exhibit distinct characteristics in other cell types, such as neutrophils^66^ or intestinal epithelial^67^ cells. Although pyroptotic cells have a morphology that clearly differentiates them from apoptotic cells, distinguishing pyroptosis from other necrotic processes, e.g., necroptosis or ferroptosis, based on morphology alone is challenging. Features like membrane integrity loss, cell rounding and swelling, and eventual PMR are common among necrotic cell death forms.^2^ Therefore, definitively identifying pyroptosis requires integrating morphological analysis with biochemical characterization and genetic knockouts. This involves studying cells or animals lacking key components of upstream pathways and gasdermins, alongside detecting active proteases and gasdermin cleavage. While inhibitors could be useful in specific contexts, no selective gasdermin inhibitors are currently available.

## Gasdermin activation and the induction of pyroptotic cell death

Expression of a full-length wild-type gasdermin cannot by itself drive pyroptosis, and requires an activation event, as summarized below:

### Activation by proteolytic cleavage

**Fig. 3:**
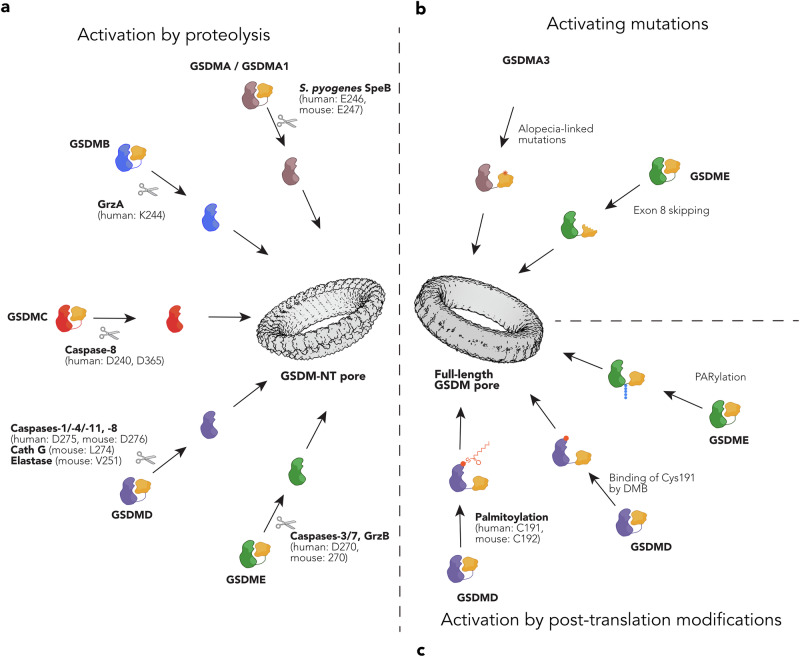
Activation of mammalian gasdermins. Three distinct mechanisms have so far been proposed to result in the activation of mammalian gasdermins. **a** Activation by proteolysis. Proteolytic cleavage within or close to the interdomain linker separates the regulatory C-terminus from the pore-forming N-terminal domain, allowing the formation of transmembrane pores by the GSDM-NT. Proteases and the respective cleavage sites are listed. **b** Activating mutations. Mutations in mouse GSDMA3 and exon skipping of human GSDME have been shown to result in a loss of autoinhibition, allowing the uncleaved protein to form membrane pores. **c** Activation by PTM. Palmitoylation of GSDMD and PARylation of GSDME have been proposed to relieve autoinhibition, thereby allowing the full-length protein to form pores. The small molecule DMB can also induce cleavage-independent GSDMD activation by binding Cys191. Gasdermin pore structures were modeled based on structures (PDB: 6VFE, and EMDB: EMD-44034) using ChimeraX1.8.

The best-studied mechanism of gasdermins is based on proteolytic cleavage and the separation of the pore-forming GSDM-NT from the regulatory GSDM-CT (Fig. 3a). Over the last decade, a number of proteases have been reported to cleave gasdermin family members and thereby initiate pore formation and cell death. Among all these proteases, caspases have emerged as the key regulators of gasdermin activation, as exemplified by the inflammatory caspases (e.g., caspase-1, -11, and -4) that in mammals cleave GSDMD.^23–25^ Interestingly though, caspase-1 can still induce pyroptosis in animals that do not feature GSDMD, such as birds, reptiles and amphibians, as it has been reported to target GSDMA under these conditions due to the presence of a caspase-1 cleavage site within its linker.^68^ A second group of caspases that target gasdermins are the apoptotic caspases, which is paradoxical as these are normally thought of as executioners of apoptosis, a non-necrotic immunologically silent form of death. For example, it has been shown that in mouse macrophages — but not human macrophages — caspase-8 cleaves GSDMD at the same residues as inflammatory caspases, and thus re-routing extrinsic apoptosis into pyroptosis, which contributes to host defense against *Yersinia*.^69–71^ Caspase-8 has also been shown to cleave GSDMC, at either D365 or D240.^72,73^ Recently a signaling axis involving ZBP1, caspase-8 and GSDMC was shown to cause pyroptosis that led to impaired mucosal repair after DSS-induced colitis in mice.^74^ Besides the initiator caspase-8, executioner caspase-3/-7 were shown to process GSDME and thereby induce pyroptosis.^75,76^ Here, the expression levels of GSDME and its potential subcellular targeting play a decisive role in determining whether a cell undergoes pyroptosis. In macrophages, for example, GSDME has been suggested to target mitochondria, promoting cytochrome c release to augment apoptosis.^77^ In cancer cell lines, the expression levels of GSDME play a role in deciding whether cells die by apoptosis or pyroptosis upon treatment with chemotherapeutic drugs, and consequently many cancer cell lines have downregulated GSDME expression.^76^ GSDME activation during apoptosis may contribute to the loss of membrane integrity in late apoptotic cells, a process known as secondary necrosis. However, it might not be the only determining factor, as NINJ1 has also been shown to permeabilize the membrane of apoptotic cells.^45^ Granzymes, a family of death-inducing proteases produced by NK cells and cytotoxic T-cells can also cleave gasdermins. For example, GrzA can process GSDMB,^78^ while GrzB processes GSDME,^79^ respectively. Thus, attack by these cells can also convert apoptosis into an inflammatory cell death if the target cell expresses the respective gasdermins. In neutrophils, GSDMD was also found to be cleaved by elastase^80^ and Cathepsin G^81^ resulting in neutrophil death. A curious case is the activation of GSDMA: to date, no mammalian enzyme has been shown to cleave GSDMA. However, SpeB, a protease from *S. pyogenes*, has been found to cleave and activate GSDMA during infection, thereby inducing pyroptosis and host defense.^82,83^

### Activation by gain-of-function mutations

The earliest reports of gasdermin-induced inflammation or cell death involve mutations that disrupt intramolecular autoinhibition, allowing the full-length protein to form pores (Fig. 3b). Notably, several mutations in mouse GSDMA3 interfere with autoinhibition interfaces I and II, leading to the protein’s autoactivation and causing alopecia in mice.^84,85^ Additionally, several gain-of-function variants of GSDME (DFNA5) have been linked to autosomal dominant hearing loss in humans.^28^ This mutated, autoactive form of GSDME arises from exon 8 skipping, which results in a truncated GSDM-CT domain and subsequent loss of autoinhibition. It is believed that the high expression of GSDME in cochlear hair cells makes them particularly vulnerable to pyroptosis triggered by mutated GSDME alleles.^30^ Mutations in DFNB59 have also been associated with hearing loss;^86^ however, since DFNB59 has not been shown to form pores, the underlying mechanism remains unclear.

### Activation by post-translational modification (PTMs)

An emerging theme is the activation of gasdermins by PTMs (Fig. 3c). So far, only three studies report cleavage-independent activation in mammals, while other examples exist for gasdermins in fungi and lower animals. For example, Du et al. recently reported that even the uncleavable GSDMD D275A mutant induced a low level of pyroptosis and IL-1β release after inflammasome activation.^87^ Further experiments linked this to ROS-dependent palmitoylation of GSDMD at C191, which is known as a PTM that promotes GSDM membrane association. However, in their study, the authors place ROS production and GSDMD palmitoylation downstream of inflammasome activation and initial GSDMD pore formation, indicating that it mainly acts as an amplification mechanism. Notably, a host of studies, however, have used D275A/D276A GSDMD as controls,^23,24^ going as far as generating a GSDMD-D276A knock-in mouse,^88^ and not found any cleavage-independent activation, indicating that in most cases cleavage is the main driver of activation. GSDMD was also reported to be activated independently of cleavage by 6,7-dichloro-2-methylsulfonyl-3-N-tert-butylaminoquinoxaline (DMB), a GLP-1 receptor agonist, which covalently modifies C191, thereby overcoming autoinhibition.^89^ Another study reported that UVC irradiation triggered DNA damage that drove activation of nuclear PARP1 and the formation of poly(ADP-ribose) polymers.^90^ Once released to the cytosol, these polymers lead to PARP5-dependent PARylation of GSDME, which induces a structural change, allowing the induction of cleavage-independent pyroptosis. Altogether the studies suggest that additional mechanisms of activation might exist and could thus expand the number of conditions or diseases in which gasdermins play a decisive role as inducers of pyroptosis.

Further insight into potential activation mechanisms comes from studies that have investigated gasdermins in lower animals and other kingdoms of life. Over the past few years, these studies have revealed that gasdermins can be found in bacteria,^91^ fungi,^92,93^ cnidaria^94^ and corals,^95^ where they have been shown to induce cell death through membrane pore formation in immunological settings such as phage defense or allorecognition. Interestingly, while processing is the primary activation mechanism of such gasdermins,^91,93^ other cleavage-independent mechanisms also exist.^94^

## Role of pyroptosis in disease

Since the discovery of the pore-forming function of gasdermins, it has become evident that pyroptosis is not solely an inflammasome/GSDMD-driven form of cell death but can also occur downstream of other gasdermins in various contexts. Since then, a growing number of studies have explored the physiological roles of gasdermins, as recently reviewed in several articles.^96,97^ Given the extensive literature on this topic, I will focus on the most well-established roles of pyroptosis in (auto)inflammatory and infectious diseases, while referring readers to excellent reviews that discuss the involvement of gasdermins and pyroptosis in cancer.^96–98^

### Role in infectious diseases

Pyroptosis is well recognized as an anti-microbial form of cell death that can restrict the growth of intracellular pathogens by killing their replicative niche. This mechanism is evident in the context of viral infections, but whether pyroptosis can kill intracellular bacteria or parasites is less well understood. A few studies have noted that gasdermins can kill intracellular bacteria,^32,99^ and thus different mechanisms have been proposed by which pyroptosis restricts bacterial replication. The antimicrobial role of pyroptosis is best understood in the intestine, where it has been shown that during infections with *Salmonella typhimurium*, cell death driven by the activation of the NLRC4 inflammasome or caspase-4 promotes the expulsion of dying cells from the intestinal epithelium,^100–102^ thereby restricting bacterial numbers within intestinal epithelial cells (IECs). It is less clear whether pyroptosis restricts bacteria in dying macrophages, but one study found that pyroptotic cell corpses can trap bacteria,^103^ which are then cleared by neutrophils that ingest the dead cells.

The anti-microbial functions of pyroptosis are mainly based on studies that explored the role of GSDMD in infectious diseases, since many of the pathogens examined are known to activate inflammasomes. These studies have reported that animals lacking inflammasome receptors, inflammatory caspases, or *Gsdmd* are highly susceptible to infections with bacteria such as *S. typhimurium*,^102^ *Burkholderia pseudomallei*,^104^ *Francisella novicida*^105^ and *Citrobacter rodentium*^23^ and *Leishmania* parasites.^106,107^ During viral infections, pyroptosis can either restrict the pathogen or cause pathology. For instance, NLRP9b and GSDMD have been shown to restrict rotavirus infections in the intestine,^108^ while NLRP3 and GSDMD-driven pyroptosis contribute to immunopathology during Norovirus infections.^109^ Additionally, H7N9 virus infection triggers a lethal cytokine storm through GSDME-mediated pyroptosis of lung alveolar epithelial cells,^110^ and GSDME-dependent pyroptosis causes severe disease during enterovirus 71 infection.^111^ Pyroptosis can also favor the pathogen during *Candida albicans* infections, where it promotes pathogen escape from infected macrophages.^112^ GSDMD is not only activated by inflammasomes but has also been reported to be cleaved by caspase-8 in *Yersinia*-infected cells. Consistently, *Gsdmd*-deficient animals have higher CFU during *Yersinia* infections.^113^ Nevertheless, some reports find that *Gsdmd*-deficient animals have no phenotype or only show a moderate increase in pathogen burden when compared to animals lacking inflammasome receptors or inflammatory caspases. This has, for example, been reported for *Legionella pneumophila* and *F. novicida*, where *Gsdmd* deficiency provided only a mild increase in CFU counts.^114,115^ A possible explanation for this discrepancy could be that in the absence of GSDMD, active caspase-1 induces a rapid lytic backup pathway that relies on active apoptotic caspases and can compensate, to some degree, for *Gsdmd* deficiency.^116,117^ It is also important to note that *Gsdmd* deficiency abrogates both pyroptosis as well as the release of IL-1β and IL-18, and thus does not allow a definitive conclusion on which inflammasome effector mechanisms drive host defense. Comparatively little is known regarding the role of other gasdermins in host defense. GSDMA has been shown to induce pyroptosis in *S. pyogenes*-infected keratinocytes, and mice deficient in *Gsdma1* or *Gsdma1*–*3* have been found to display larger lesions and uncontrolled bacterial dissemination to systemic sites.^82,83^

GSDME activation can also mediate anti-microbial immunity. Infections with VSV-1 or HSV-1 activate intrinsic apoptosis, which in keratinocytes results in GSDME-dependent pyroptosis that releases IL-1α and restricts infection.^118^ Similarly, induction of extrinsic apoptosis, induced by *Yersinia* infections, results in GSDME activation in neutrophils and the initiation of a host defence.^113^ While gasdermin activation is also detrimental in models of septic shock, *Gsdmd* deficiency, for example, was found to be protective in LPS-induced lethality models^23^ and in polymicrobial sepsis caused by cecal ligation and puncture (CLP).^119^ Gasdermin-dependent PS exposure has also been shown to trigger blood clotting and tissue thrombosis upon LPS injection in mice,^120^ and GSDMD-dependent pyroptosis in endothelial cells causes the disruption of the blood–brain barrier during LPS challenge or *Klebsiella pneumoniae* infections.^121^ These data suggest that inhibiting inflammasomes or GSDMD in sepsis could be beneficial for patients.

### Autoinflammatory genetic diseases

Aberrant activation of inflammasomes causes or contributes to multiple hereditary autoinflammatory diseases. Gain-of-function mutations in NLRP3 for example cause a spectrum of diseases, such as familial cold urticaria (FCAS), Muckle–Wells syndrome (MWS), and neonatal-onset multisystem inflammatory disease (NOMID, also referred to as chronic infantile neurological cutaneous and joint syndrome, CINCA), that are commonly referred to as cryopyrin-associated periodic syndromes (CAPS) or NLRP3-associated autoinflammatory diseases (NLRP3-AID).^122^ The role of GSDMD has been experimentally addressed in a mouse model of NOMID, and shown to prevent the development of symptoms, suggesting that GSDMD pores are a main driver of disease.^123^ Yet, since most NLRP3-AID patients are highly responsive to IL-1β-targeted therapies, it indicates that not pyroptosis per se, but the associated cytokine release drives disease. Another disease caused by mutations in inflammasome receptors, is Familial Mediterranean Fever (FMF), which is the most prevalent monogenic autoinflammatory disease worldwide and caused by mutations in MEFV (encoding the protein Pyrin). GSDMD deficiency has also been shown to abrogate auto-inflammation in the context of an animal model of FMF (expressing MefvV726A).^124^

## Therapeutic targeting of pyroptosis

Given the role of gasdermins and pyroptosis in human disease, the development of drugs that inhibit pyroptosis could open new avenues for anti-inflammatory therapies. Anti-IL-1 therapies are currently used with success to reduce inflammation in patients affected by inflammasomopathies.^125^ However, these therapies have shown limited impact on patients with NOMID, suggesting that IL-1 may not be the primary driver of disease in this context. This highlights the potential benefit of targeting upstream processes. In this regard, the ongoing development of inhibitors that directly target NLRP3, such as MCC950,^126^ could prove valuable.

**Fig. 4:**
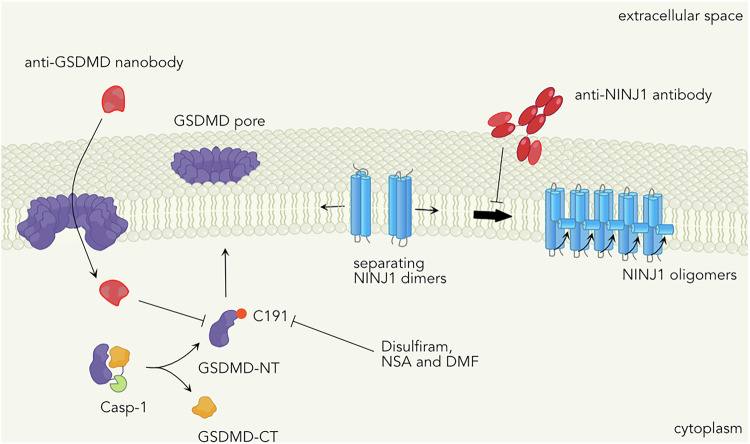
Inhibition of pyroptosis at the level of GSDMD or NINJ1. Treatment with Dimethyl fumarate (DMF), Necrosulfonamide (NSA) and Disulfirma have been shown to block GSDMD pore formation by targeting C191/C192 in human and mouse GSDMD, respectively. Anti-GSDMD antibodies can also block GSDMD pore formation but require entry into the cell through already existing GSDMD pores. Antagonizing NINJ1 oligomerization with antibodies can prevent the lysis step following GSDMD pore formation.

Inhibition of GSDMD or other gasdermins represents an alternative approach that could reduce both inflammasome-associated cell death and cytokine release. Several potential inhibitors have been reported over the years, including Dimethyl fumarate (DMF),^127^ necrosulfonamide,^128^ and disulfiram^129^ (Fig. 4). The mechanism of these drugs is likely linked to the crucial role of palmitoylation at Cys191/192 in the membrane association of cleaved GSDMD-NT.^87,130^ For example, DMF induces succination of Cys192,^127^ among other residues, while necrosulfonamide and disulfiram also target Cys192,^128,129^ reducing its palmitoylation.^87^ Interestingly, DMF also blocks GSDME,^127^ as it succinates Cys45, suggesting a similar mechanism.

While these drugs may not be specific to GSDMD alone, animal models of LPS-induced lethality, Mediterranean fever, and experimental autoimmune encephalitis have shown that they can block inflammasome- or pyroptosis-induced inflammation in vivo.^127,129^ Another alternative could be the use of antibodies that target GSDMD (Fig. 4). Recent studies have demonstrated that administration of antagonistic GSDMD nanobodies can prevent pyroptosis and cytokine release in cell culture,^131^ and shown that this involves an entry of these nanobodies through GSDMD pore, which then prevent further GSDMD pore formation. However, whether this approach would be effective in vivo remains to be tested.

An alternative approach to preventing inflammation associated with pyroptosis could involve inhibiting cell lysis and DAMP release driven by NINJ1 (Fig. 4). Two recent studies have reported a role for NINJ1 in models of liver damage in mice, caused by LPS plus D-galactosamine, concanavalin A, Jo2 anti-Fas agonist antibody, or ischemia-reperfusion injury.^132,133^ One of these studies also demonstrated that anti-NINJ1 neutralizing antibodies, which prevent NINJ1 oligomerization, significantly reduced circulating DAMPs, including IL-18, HMGB1, mitochondrial DNA, and markers of liver inflammation.^133^ However, further characterization of NINJ1 in vivo is needed to fully assess its physiological significance. Interestingly, no role for NINJ1 was found in a model of LPS-induced septic shock, which has been shown to be GSDMD-dependent.^23^ Thus, additional experiments are required to validate NINJ1 as a potential target for therapeutic interventions.

## Perspectives

Since the discovery of pyroptosis, significant progress has been made in understanding the molecular mechanisms that induce this form of cell death, as well as its role in health and disease. The groundbreaking identification of gasdermins as the executioners of pyroptosis has transformed the field, providing insight into how cells undergo pyroptosis and enabling genetic studies to explore the importance of gasdermins and pyroptosis in various diseases. These studies have demonstrated that pyroptotic cell death is a physiologically significant process essential for host defense against a wide range of pathogens. However, it has also become clear that when pyroptosis occurs uncontrollably, as seen in inflammasomopathies, it can be highly detrimental to the host, underscoring the need for strategies to regulate pyroptosis induction.

To address this, further research is needed to explore how cells control gasdermin activation, particularly through PTMs and other mechanisms that govern gasdermin–membrane interactions and pore formation. Understanding these processes could uncover innovative strategies to block pyroptosis. Additionally, research should focus on developing inhibitors or antagonistic antibodies. These have shown promise, as anti-GSDMD nanobodies can reduce pyroptosis in cells, and anti-NINJ1 antibodies can alleviate inflammation in liver injury models. It is crucial to further validate these approaches in disease models where gasdermins are confirmed to be involved. Given the multifaceted roles that gasdermins play in inflammation and cell death, the quest to develop new ways to modulate their activity will undoubtedly remain a dynamic area of research in the years ahead.
